# Sanitary Commission

**Published:** 1863-05

**Authors:** 


### SANITARY COMMISSION.

This Commission has been doing an excellent work in the Army of the Potomac. A correspondent of the Philadelphia *Inquirer* writes as follows:

"Attached to the Commission are Medical Inspectors, highly educated physicians, whose duty it is to visit the several regiments and inspect the hospital, the camp, and the surroundings of each regiment, and report as to its healthfulness and general condition for cleanliness and good order.

In addition to these Inspectors are Relief Agents, who dispense to the hospitals, and sick soldiers in quarters, articles of clothing, delicacies, &c., not furnished by the Government, but really necessary for the sick and convalescent.

The General Superintendent of the operations of the Commission in the Army is Dr. Isaac N. Kerlin, formerly connected with the Pennsylvania Training School for Feeble-Minded Children, at Media, Delaware County, Pa. The doctor is a most active and energetic officer, and the affairs of the Commission could not have been entrusted to more able hands. The Inspector for the First and Sixth Corps is Dr. A. McDonald, and Mr. W. Murray, Relief Agent. For Second, Third and Fifth Corps, Dr. F. W. Johnston is the Inspector, and Rev. Wm. Harris and Mr. Edward Abbott the Relief Agents. For Eleventh and Twelfth Corps, Dr. W. F. Swalm is the Inspector, and James Gall, jr., the Relief Agent. These gentlemen make daily visits in the several corps, and promptly respond to all requisitions made upon them for supplies for the sick and suffering.

The Commission has two store tents at Falmouth depot, and a storehouse at Aquia Creek, and their goods are brought down from Washington in a steamboat of their own, thus putting the Government to no expense for transportation. At Aquia Creek they have a Lodge, superintended by Mr. E. Fay, where sick and discharged soldiers are accommodated with meals and lodgings when detained at Aquia waiting to take the boat for Washington. In Washington the Commission have also "Lodges," where sick and discharged soldiers are accommodated free of charge until they receive their pay from the Government, and the officers of the Commission also afford the poor soldier every facility and advice to secure his pay without resorting to sharpers and claim agents.

Besides these works of active benevolence, the Commission have secured through the labors of their inspecting officers an immense mass of information of the greatest value to the scientific world, relating to sickness and mortality incident to camp life—the character, arrangement, cleanliness and condition of camps and their sites; the source and quality of the water used by the men, and its effect upon their health; the rations and cooking, and the general discipline of each body inspected. The Commission is invested with a semi-official authority, having the power to report violations or neglect of duty on the part of surgeons; and when a hospital is found in bad condition, a report of the fact by the Inspectors of the Sanitary Commission is sure to have the evil remedied immediately by the Surgeon-General or the Medical Director of the corps to which the regiment may be attached."—*Surgical Reporter.*

